# Angiotensin II Type II Receptor Deficiency Accelerates the Development of Nephropathy in Type I Diabetes via Oxidative Stress and ACE2

**DOI:** 10.1155/2011/521076

**Published:** 2011-10-27

**Authors:** Shiao-Ying Chang, Yun-Wen Chen, Isabelle Chenier, Stella Le Minh Tran, Shao-Ling Zhang

**Affiliations:** Research Centre, Centre Hospitalier de l'Université de Montréal (CRCHUM), Hôtel-Dieu, Pavillon Masson, 8-227, 3850 Saint Urbain Street, Montréal, QC, Canada H2W 1T7

## Abstract

Since the functional role(s) of angiotensin II (Ang II) type II receptor (AT_2_R) in type I diabetes is unknown, we hypothesized that AT_2_R is involved in decreasing the effects of type I diabetes on the kidneys. We induced diabetes with low-dose streptozotocin (STZ) in both AT_2_R knockout (AT_2_RKO) and wild-type (WT) male mice aged 12 weeks and followed them for 4 weeks. Three subgroups nondiabetic, diabetic, and insulin-treated diabetic (Rx insulin implant) were studied. Systolic blood pressure (SBP), physiological parameters, glomerular filtration rate (GFR), renal morphology, gene expression, and apoptosis were assessed. After 4 weeks of diabetes, compared to WT controls, AT_2_RKO mice clearly developed features of early diabetic nephropathy (DN), such as renal hypertrophy, tubular apoptosis, and progressive extracellular matrix (ECM) protein accumulation as well as increased GFR. AT_2_RKO mice presented hypertension unaffected by diabetes. Renal oxidative stress (measured as heme oxygenase 1 (HO-1) gene expression and reactive oxygen species (ROS) generation) and intrarenal renin angiotensin system components, such as angiotensinogen (Agt), AT_1_R, and angiotensin-converting enzyme (ACE) gene expression, were augmented whereas angiotensin-converting enzyme2 (ACE2) gene expression was decreased in renal proximal tubules (RPTs) of AT_2_RKO mice. The renal changes noted above were significantly enhanced in diabetic AT_2_RKO mice but partially attenuated in insulin-treated diabetic WT and AT_2_RKO mice. In conclusion, AT_2_R deficiency accelerates the development of DN, which appears to be mediated, at least in part, via heightened oxidative stress and ACE/ACE2 ratio in RPTs.

## 1. Introduction

Diabetic nephropathy (DN) is the single major cause of end-stage renal failure in North America [1, 2]. Among the multiple risk factors contributing to diabetic renal disease, the renin-angiotensin system (RAS), a coordinated hormonal cascade that has major physiological and pathological effects on the cardiovascular and renal functions, is one of the most important systems affecting DN development and progression [3–5]. 

Although chronic treatment with RAS blockers is effective in controlling hypertension and retarding DN progression, it is not a cure, indicating that the mechanisms of renal protection by RAS blockers in diabetes are far from being completely understood, and the discovery of additional therapeutic pathways as potential drug targets is of paramount importance [2, 6].

Intrarenal angiotensin II (Ang II), a principal effector of the RAS that is increased in DN, acts through 2 major angiotensin receptors, Ang II type I receptor (AT_1_R) and AT_2_R, and may be involved in dysfunctional responses in the diabetic kidney [3–5]. For instance, Ang II effects within the kidney are mediated by AT_1_R and include cellular dedifferentiation and proliferation, renal hypertrophy and apoptosis, vasoconstriction and heightened renal and tubular sodium resorption. In contrast to the well-established functional impact of AT_1_R in DN, the functional role of AT_2_R in the development of nephropathy in type I diabetes is incompletely understood. AT_2_R activation appears to suppress renin biosynthesis and release from renal juxtaglomerular cells, leading to vasodilatation and natriuresis, both of which may reduce blood pressure (BP) [3–5]. 

In the kidneys, AT_2_R expression in both embryonic and mature stages (i.e., glomerular endothelial cells, podocytes, tubular epithelial cells, and inner medullary collecting ducts) is known and well defined [7]. AT_2_R-deficient mice (i.e., AT_2_R knockout, AT_2_RKO) display a phenotype similar to that of humans with congenital renal and urinary tract anomalies [8, 9] and incur hypertension in adulthood [8]. These phenotypes highlight the importance of AT_2_R in normal kidney development and BP regulation, but understanding how AT_2_R is regulated remains elusive.

An additional component of the RAS family, angiotensin-converting enzyme2 (ACE2) [10, 11], shares 42% homology with angiotensin-converting enzyme (ACE) but has different biochemical activities. ACE2 specifically cleaves Ang I and Ang II into Ang 1-9 and Ang 1-7, respectively. Since ACE2 and ACE are coexpressed in many tissues, and ACE2 expression is both cardio- and renoprotective, acting in a counterregulatory manner to ACE in both experimental animals and humans, alterations in their activities and the ACE/ACE2 ratio may participate in hypertension and renal disease [12–14]. Whether AT_2_R and ACE2 interact in diabetes, including DN, is unknown. However, since AT_2_R inhibits ACE activity [15], we speculated that AT_2_R in diabetes might lead to heightened ACE2 activity and then to an increased ACE2/ACE ratio, which could be important in vasodilatation and natriuresis in DN. 

In the current *in vivo* study, we examined the potential functional role(s) of AT_2_R deficiency in nephropathy development in type I diabetes. We hypothesized that AT_2_R deficiency will accelerate the progression of DN in type I diabetes via reactive oxygen species (ROS) generation and upregulation classic intrarenal RAS gene expression with downregulation of ACE2 gene expression in renal proximal tubules (RPTs).

## 2. Methods

### 2.1. Animals

Male wild-type (WT, C57/BL6) and AT_2_RKO mice (C57/BL6 background, obtained from Dr. Tadashi Inagami, Department of Biochemistry, Vanderbilt University School of Medicine, Nashville, Tenn, USA) [8] were studied* in vivo*. 

As described previously [16–18], we induced diabetes in male AT_2_RKO and WT mice at the age of 12 weeks with intraperitoneal multiple injections of streptozotocin (STZ, Sigma-Aldrich Canada Ltd., Oakville, ON, Canada) at a low-dose level of ~45–50 mg per kg body weight (BW) daily for 5 consecutive days. Three subgroups (nondiabetic (control), diabetic, and diabetic treated with insulin implants (LinShin Canada, Inc., Toronto, ON, Canada)) were sacrificed at the age of 17 weeks after a 4-week experimental period. 

Animal care in these experiments met the standards set forth by the Canadian Council on Animal Care, and all procedures were approved by the Institutional Animal Care Committee of the CRCHUM. All AT_2_RKO and WT (C57/BL6) mice were housed under standard conditions of humidity and lighting (12-hour light-dark cycles), with free access to standard mouse chow and water* ad libitum*.

### 2.2. Physiological Studies

Blood glucose levels were measured with a Side-Kick Glucose Analyzer (Model 1500, Interscience, ON, Canada) after a 4-hour fast in the morning as reported previously [16–18]. Mean systolic blood pressure (SBP) was monitored by the tail-cuff method with the Visitech BP-2000 Blood Pressure Analysis System for mice (Visitech System Inc., Apex, NC, USA) as reported elsewhere [16–18]. Briefly, all animals were conditioned and acclimated for 2 weeks (20–30 min of SBP measurements per session, thrice weekly) starting at 10 weeks of age, and then SBP was measured thrice weekly and averaged per week from 12 weeks of age until 16-weeks of age. Since the animals were acclimated to BP measurement, we judged that the stress of having SBP recorded by tail cuff was relatively minimized. While the technique of tail-cuff measurement is generally considered less sensitive than telemetry, we believe that our SBP data is valid and convincing, based on the substantial numbers of animals used and the longitudinal studies.

All animals were euthanized at 17 weeks of age under CO_2_ and the kidneys removed immediately. Body weight (BW) and kidney weight (KW) were rapidly recorded. The left kidney was utilized for renal morphology and immunohistochemistry (IHC). The right kidney was reserved for renal proximal tubule (RPTs) isolation by the Percoll gradient method as well as for gene expression experiments as previously reported [16–18].

### 2.3. Glomerular Filtration Rate (GFR) Measurement

We estimated the GFR in 16 weeks old animals according to the protocol described by Qi et al. [19] and as recommended by AMDCC (http://www.diacomp.org/). In brief, each mouse received a single intravenous bolus of 5% fluorescein isothiocyanate-inulin (FITC-inulin), after which 7 blood samples (each ~20 *μ*L) were collected from the saphenous vein at 3, 7, 10, 15, 35, 55, and 75 min post-FITC-inulin injection. Plasma fluorescence concentration at each time point was measured by Fluoroscan Ascent FL (Labsystems, Helsinki, Finland) with 485 nm excitation and read at 538 nm emission. The GFR was calculated according to the equation: GFR = *I*/(*A*/*α* + *B*/*β*), where *I* was the amount of FITC-inulin bolus delivered, *A* and *α* were the *y *intercept and decay constant of the rapid (initial) decay phase, respectively, and *B* and *β* were the *y *intercept and decay constant of the slow decay phase, respectively [19].

### 2.4. Renal Morphology

Paraffin-embedded renal sections (4 to 5 specimens per group) were stained with Periodic Acid Schiff (PAS) and Masson's Trichrome staining and visualized by light microscopy by an observer blinded to the treatment group. The collected glomerular images were analyzed and quantified by NIH Image J software (http://rsb.info.nih.gov/ij/) as reported previously [20–22].

### 2.5. Apoptosis Assay

Apoptosis was quantified by the transferase-dUTP-nicked-end labeling (TUNEL) as reported previously [16, 23]. Semiquantitation of apoptotic cells in the kidneys was performed as previously reported [20–22].

### 2.6. ROS Generation

Freshly isolated RPTs were immediately processed for ROS measurement by the lucigenin method as described previously [20, 21, 24]. ROS production was normalized with protein concentration and expressed as relative light units (RLUs) per *μ*g protein.

### 2.7. Real-Time-Quantitative Polymerase Chain Reaction (RT-qPCR)

Total RNA extracted from freshly isolated RPTs was assayed for gene expression by real time quantitative PCR (RT-qPCR) as reported previously [16, 17, 23]. Fast SYBR Green Mastermix kit and the 7500 fast real-time PCR system (Applied Biosystems, Life Technologies, Foster City, Calif, USA) were employed for this purpose.

### 2.8. Immunohistochemistry

Immunohistochemistry (IHC) was performed by the standard avidin-biotin-peroxidase complex method (ABC Staining System, Santa Cruz Biotechnologies, Santa Cruz, Calif, USA), as described elsewhere [16, 23]. Polyclonal anti-angiotensinogen (Agt) antibody was a gift from Dr. John S.D. Chan (CRCHUM—Hôtel-Dieu Hospital). Other antibodies, including AT_1_R, ACE, and anti-heme oxygenase-1 (HO-1), were purchased from Santa Cruz Biotechnologies. Monoclonal ACE2 antibody was procured from R&D Systems, Inc. (Burlington, ON, Canada).

### 2.9. Statistical Analysis

Statistical significance between the experimental groups was analyzed by 1-way ANOVA, followed by the Bonferroni test with Graphpad Software, Prism 5.0 (http://www.graphpad.com/prism/Prism.htm). A probability level of *P ≤* 0.05 was considered to be statistically significant as compared with WT control animal.

## 3. Results

### 3.1. Physiological Findings

**Figure 1:**
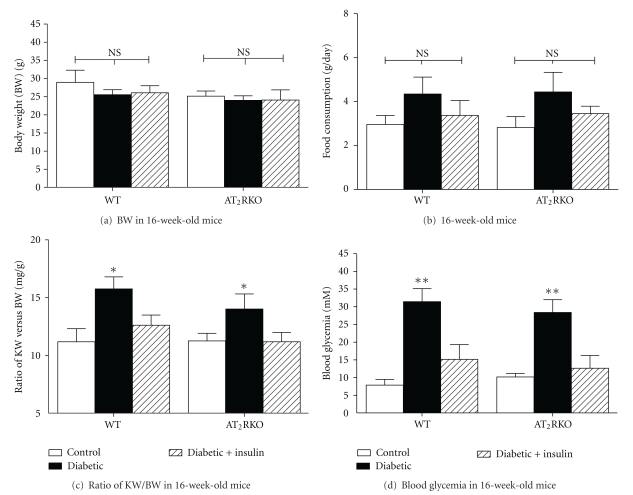
Physiological parameters measurement in 3 groups (control (open bar), diabetic (black bar), and insulin-treated diabetic (shadowed bar)) of both WT and AT_2_RKO male mice at the age of 16 weeks. (a) Gross body weight (BW, g); (b) Food consumptions (g/day); (c) KW/BW ratio; (d) blood glycemic concentration (mM). *P ≤ 0.05; **P ≤ 0.01; NS, non significant.

We measured some biological parameters such as BW (g), KW (mg), daily food consumption (g/day), and blood glycemic concentration (mM) in 3 subgroups of animals (nondiabetic control, and diabetic, with or without insulin treatment) of both WT and AT_2_RKO mice. There were no significant differences between AT_2_RKO and WT mice with or without diabetes in terms of BW (Figure 1(a)) (WT mice (g), control (*N* = 15), 28.9 ± 3.39; diabetic (*N* = 14), 25.52 ± 1.38; diabetic with insulin treatment (*N* = 13), 26.07 ± 1.91; AT_2_RKO mice (g), control (*N* = 15), 25.13 ± 1.40; diabetic (*N* = 15), 23.96 ± 1.25; diabetic with insulin treatment (*N* = 13), 24.05 ± 2.82 and daily food consumption (Figure 1(b)) (WT mice (g/day), control (*N* = 15), 2.96 ± 0.41; diabetic (*N* = 14), 4.35 ± 0.77; and diabetic with insulin treatment (*N* = 13), 3.37 ± 0.67; AT_2_RKO mice (g), control (*N* = 15), 2.82 ± 0.50; diabetic (*N* = 15), 4.44 ± 0.89; diabetic with insulin treatment (*N* = 13), 3.45 ± 0.34). As expected, renal hypertrophy (Figure 1(c)) (defined by the ratio of KW versus BW) was present in diabetic animals of both AT_2_RKO and WT mice; insulin therapy normalized the hyperglycemia (Figure 1(d)) (WT mice (mM), control (*N* = 15), 7.87 ± 1.59; diabetic (*N* = 14), 31.43 ± 3.68; diabetic with insulin treatment (*N* = 13), 15.13 ± 4.18; AT_2_RKO mice (mM), control (*N* = 15), 10.16 ± 0.99; diabetic (*N* = 15), 28.38 ± 3.6; diabetic with insulin treatment (*N* = 13), 12.64 ± 3.59). 

### 3.2. Mean Systolic Blood Pressure (SBP) and Glomerular Filtration Rate (GFRs) Measurement

**Figure 2:**
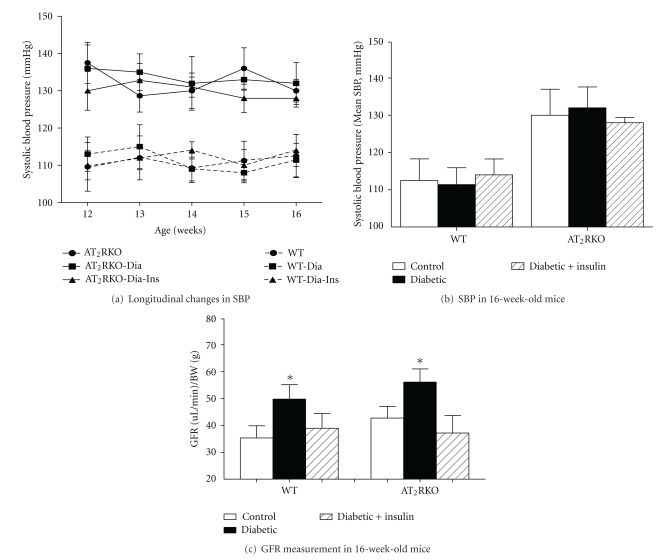
(a) Longitudinal and cross-sectional changes in mean SBP in 3 subgroups (e.g., control (●), diabetic (■), and insulin-treated diabetic (▲)) of WT (broken line) and AT_2_RKO male mice (solid line) from the age of 12 to 16 weeks. (b) Mean SBP in 3 groups control (open bar), diabetic (black bar), and insulin-treated diabetic (shadowed bar) of WT and AT_2_RKO male mice at the age of 16 weeks. (c) GFR in 3 groups (control (open bar), diabetic (black bar), and insulin-treated diabetic (shadowed bar)) of WT and AT_2_RKO male mice at age 16 weeks. *P ≤ 0.05.

SBP increased over time in AT_2_RKO mice as reported [8] (Figure 2(a)), and the development of hypertension appeared independent of diabetes (Figure 2(b)). For example, after 4 weeks of diabetes, there was no further increase in the SBP in either WT or AT_2_RKO mice (Figure 2(b)) (WT mice, control (*N* = 10), 112.5 ± 5.78 mmHg; WT diabetic (*N* = 12), 111.40 ± 4.51 mmHg; WT diabetic with insulin treatment (*N* = 9), 114 ± 4.27 mmHg; AT_2_RKO mice, control (*N* = 13), 130.04 ± 7.00 mmHg; AT_2_RKO diabetic (*N* = 12), 132.20 ± 5.62 mmHg; AT_2_RKO diabetic with insulin treatment (*N* = 10), 128.97 ± 1.28 mmHg).

AT_2_RKO mice had higher GFRs [(uL/min)/BW(g)] by FITC-inulin measurement (Figure 2(c)) (control: 42.74 ± 4.3, (*N* = 8); diabetic 56.10 ± 4.9, (*N* = 8); diabetic with insulin treatment 37.16 ± 6.5, (*N* = 7)) at 16 weeks of age as compared to WT (control: 35.33 ± 4.5, (*N* = 8); diabetic 49.78 ± 5.4, (*N* = 9); and diabetic with insulin treatment 38.93 ± 5.56, (*N* = 7)). Thus, renal hyperfiltration occurs in AT_2_RKO mice, which may possibly contribute to the development of hypertension.

### 3.3. Renal Morphology and Apoptosis

**Figure 3:**
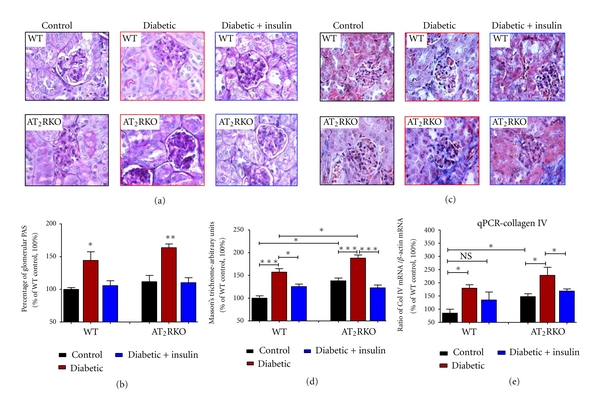
Renal morphology assessed by (a) and (b) PAS staining and (c) and (d) Masson's trichrome staining (magnification 600X). (b) and (d) Quantification of relatively stained arbitrary units of PAS (b) and Masson's Trichrome (d) in 3 subgroups [control (black bar), diabetic (red bar) and insulin-treated diabetic (blue bar)] of WT and AT_2_RKO male mice at the age of 16 weeks. The *y*-axis shows the percentage of relative staining values compared to WT control animals (100%). (e) Renal collagen IV mRNA expression analyzed by RT-qPCR. Quantitation of renal collagen IV gene was normalized to its own *β*-actin mRNA. The *y*-axis shows the percentage of relative values compared to WT animals (100%). *P ≤ 0.05; **P ≤ 0.01; ***P ≤ 0.001; NS, nonsignificant.

**Figure 4:**
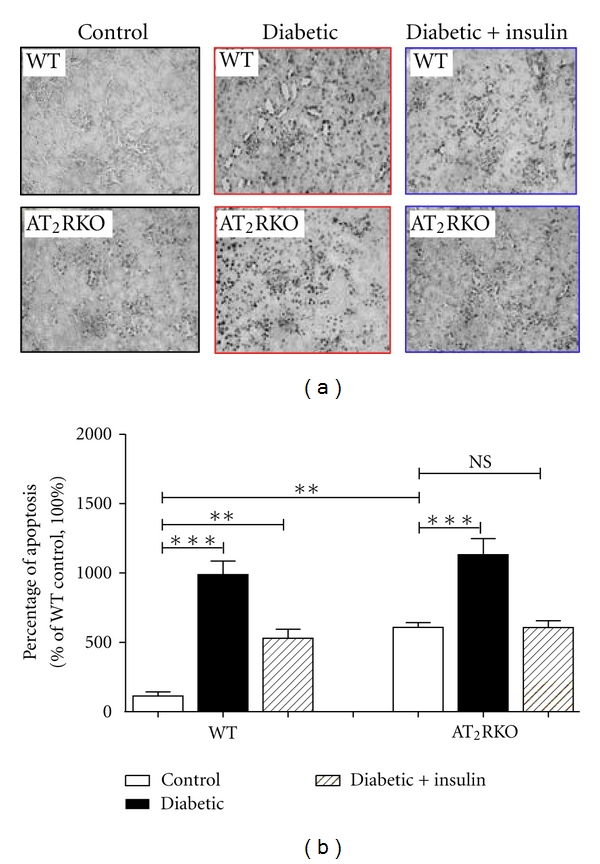
(a) TUNEL assay (magnification 200X) in the kidneys of 3 subgroups [control (open bar), diabetic (black bar) and insulin-treated diabetic (shadowed bar)] of WT and AT_2_RKO male mice at age 16 weeks. (b) Semi-quantitation of apoptotic cells in the kidneys. The *y*-axis shows the fold increase of apoptotic cell number in the kidneys of 3 subgroups of mice. **P ≤ 0.01; ***P ≤ 0.001; NS, non-significant.

Extracellular matrix (ECM) protein accumulation in glomeruli and tubular apoptosis are the key features of diabetic nephropathy. As compared to WT animals at the age of 16 weeks, AT_2_RKO mice display increased ECM accumulation in the glomeruli (Figures 3(a)–3(b), PAS staining) and tubulointerstitium (Figures 3(c) and 3(d), Masson trichrome staining) with upregulation of renal collagen IV mRNA level (Figure 3(e)), as well as tubular apoptosis (Figure 4, TUNEL assay). These features were further pronounced in diabetic animals and partially attenuated in the insulin-treated groups in both WT and AT_2_RKO mice. 

### 3.4. ROS Generation

**Figure 5:**
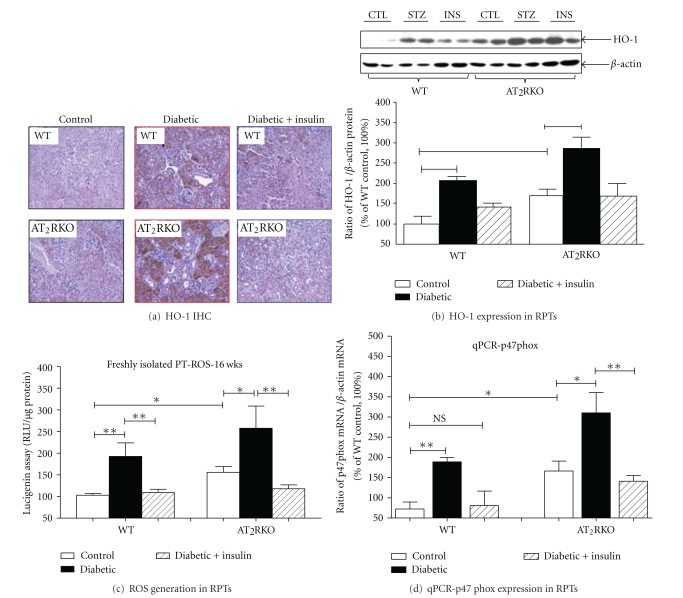
(a) HO-1 IHC staining (magnification 200X) in the kidneys of 3 subgroups (control, diabetic, and insulin-treated diabetic) of both WT and AT_2_RKO male mice. (b) HO-1 expression analyzed by Western blot in freshly isolated RPTs from 3 subgroups (control (open bar), diabetic (black bar), and insulin-treated diabetic (shadowed bar)) of WT and AT_2_RKO male mice. The quantitation of the relative densities of HO-1 normalized to *β*-actin. The *y*-axis shows the percentage of relative values compared to WT controls (100%). **P ≤ 0.01; (c) ROS measurement in RPTs. The *y*-axis shows relative ROS production values compared to WT controls (100%). (d) p47phox mRNA expression in RPTs analyzed by RT-qPCR. Quantitation of p47phox gene was normalized to its own *β*-actin mRNA. The *y*-axis shows the percentage of relative values compared to WT animals (100%). *P ≤ 0.05. **P ≤ 0.01; NS, nonsignificant.

Heme oxygenase 1 (HO-1) is an oxidative stress-inducible enzyme that confers cellular oxidative stress *in vivo,* as reported previously [25]. Compared to WT mice at the age of 16 weeks, HO-1 expression, as seen by IHC (Figure 5(a)) and Western blot (Figure 5(b)), was increased significantly in RPT cells of AT_2_RKO mice, and this increment was even more marked in diabetic and prevented in insulin-treated AT_2_RKO mice. 

We confirmed the renal HO-1 IHC results in freshly isolated RPTs from the 3 subgroups of AT_2_RKO and WT mice by measuring ROS generation according to the lucigenin method [20, 21, 24]. Compared to WT animals, ROS generation was significantly augmented in freshly isolated RPTs from AT_2_RKO mice (Figure 5(c)), and this ROS elevation was even more striking in diabetic and attenuated in insulin-treated AT_2_RKO mice (Figure 5(c)). Moreover, as compared with control WT mice, p47phox mRNA (one of NADPH oxidase components) seems to be upregulated in RPTs of AT_2_RKO mice, and this upregulation appears to be more profound in diabetic condition (Figure 5(d)).

### 3.5. Activation of Intrarenal RAS

**Figure 6:**
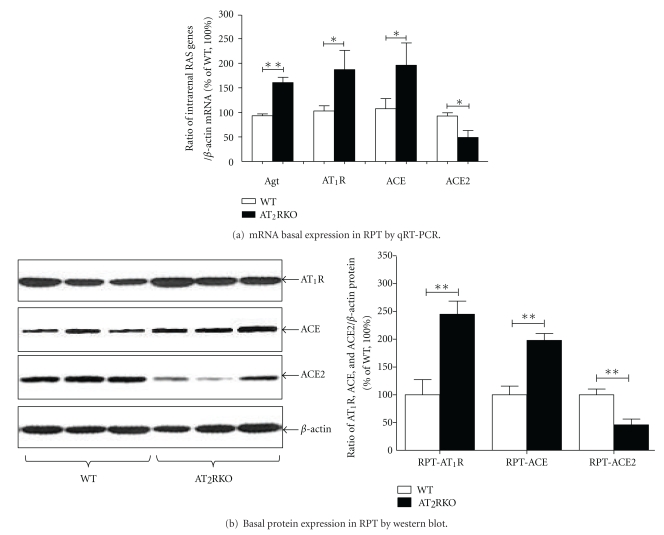
Basal expression of intrarenal RAS genes (i.e., Agt, AT_1_R, ACE, and ACE2) in freshly isolated RPTs from WT (open bar) and AT_2_RKO (black bar) male mice at the age of 16 weeks. (a) RT-qPCR. Quantitation of relative intra-renal RAS genes was normalized to their own *β*-actin mRNA. The *y*-axis shows the percentage of relative values compared to WT animals (100%). *P ≤ 0.05. (b) Western blotting. Quantitation of relative densities of intrarenal RAS genes was normalized to *β*-actin. The *y*-axis shows the percentage of relative values compared to WT animals (100%). **P ≤ 0.01.

First, we assessed the basal expression of several key components of the RAS—Agt, AT_1_R, ACE, and ACE2—by RT-qPCR and Western blotting in freshly isolated RPTs from both WT and AT_2_RKO mice. Compared to WT animals, the intrarenal RAS genes, that is, Agt, AT_1_R, and ACE, were increased, but ACE2 was decreased in RPTs of AT_2_RKO mice (Figure 6).

**Figure 7:**
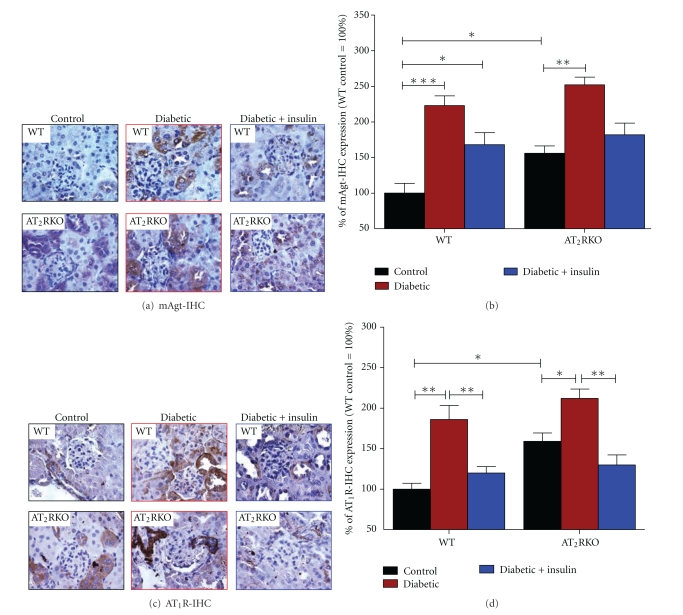
IHC staining of mAgt (a) and (b) and AT_1_R (c) and (d) genes. (a) and (c) IHC images of Agt and AT_1_R in the kidneys of 3 subgroups of both WT and AT_2_RKO male mice (magnification 600X). (b) and (d) Semiquantification of IHC expression in the kidneys of 3 subgroups (control (black bar), diabetic (red bar), and insulin-treated diabetic (blue bar)) of WT and AT_2_RKO male mice. The* y*-axis shows the percentage of relative staining values compared to WT control animals (100%). *P ≤ 0.05; **P ≤ 0.01; ***P ≤ 0.001.

**Figure 8:**
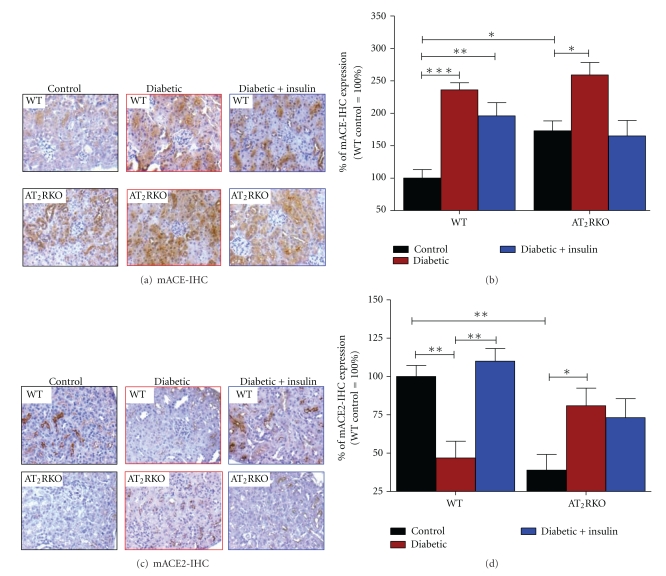
IHC staining of ACE (a) and (b) and ACE2 (c) and (d) genes. (a) and (c) IHC images of ACE and ACE2 in the kidneys of 3 subgroups of WT and AT_2_RKO male mice (magnification 200X). (b) and (d) Semiquantification of IHC expression in the kidneys of 3 subgroups (control (black bar), diabetic (red bar), and insulin-treated diabetic (blue bar)) of WT and AT_2_RKO male mice. The *y*-axis shows the percentage of relative staining values compared to WT controls (100%). *P ≤ 0.05; **P ≤ 0.01; ***P ≤ 0.001.

To establish a linkage between nephropathy development and intrarenal RAS activation in diabetes more completely, we evaluated intrarenal RAS genes by IHC in the kidneys of 3 subgroups of animals from both AT_2_RKO and WT mice. As compared to WT mice, the enhanced Agt, AT_1_R, and ACE were more striking in the diabetic kidneys of AT_2_RKO. For example, the elevation of Agt protein expression was mainly localized in the proximal tubules (Figures 7(a) and 7(b)). AT_1_R were detected in the proximal tubules and small intrarenal vessels (Figures 7(c) and 7(d)) with ACE on the luminal side of the proximal tubules (Figures 8(a) and 8(b)). The increased Agt, AT_1_R, and ACE expression appeared to be partially ameliorated in the kidneys of insulin-treated animals (Figure 7 and Figure 8). 

In contrast, ACE2 gene expression was significantly downregulated in RPTs of AT_2_RKO mice (Figure 6). Although its expression was similar in location to that of ACE, ACE2 was significantly decreased in RPTs of AT_2_RKO mice, but was relatively increased in diabetic kidneys of AT_2_RKO mice (Figures 8(c) and 8(d)), consistent with previously reported counterregulatory interactions between ACE and ACE2, which may play a role in hypertension in diabetes [13, 14, 26].

## 4. Discussion

The present study examined potential mechanisms of the functional role(s) of AT_2_R deficiency in the development of nephropathy in type I diabetes. AT_2_R deficiency accelerated the development of DN, which appears to be mediated, at least in part, via elevated oxidative stress and ACE/ACE2 ratio in RPTs.

In contrast to the well-established functional impact of AT_1_R in DN, few data are currently available concerning the role of the AT_2_R in diabetes and DN progression. It has been postulated that the action of AT_2_R may counterbalance the action of AT_1_R in hypertension and kidney disease. For instance, activation of the AT_2_R leads to vasodilatation [27] (e.g., possibly via activation of bradykinin-cGMP-nitric oxide (NO) pathway [28] and suppression of renin biosynthesis and release from renal juxtaglomerular cells [28]; these lead to vasodilatation and natriuresis, which then reduces blood pressure), mediates natriuresis (e.g., possibly via the renal dopaminergic system, a crucial system in the control of renal sodium excretion and blood pressure) [29], and causes antiproliferative/proapoptotic responses [30]. Thus, it seems reasonable that AT_2_R might have a counterregulatory role opposing AT_1_R-mediated vasoconstriction, but such mechanistic conclusions are based mainly on the studies in which an antagonist of AT_2_R, PD123319, was used to block AT_2_R function [29]. In this regard, a model in which AT_2_R is ablated, such as AT_2_RKO mice, has been eagerly used to determine whether activation of the AT_2_R is important in the progression of diabetic nephropathy. If so, manipulating the AT_2_R could act as a potential therapeutic strategy in DN. 

Our studies confirmed observations that, as compared to WT animals, AT_2_RKO enhanced renal fibrosis [31]. After 4 weeks of diabetes, AT_2_RKO develops clear evidence of DN, showing renal hypertrophy, tubular apoptosis, and progressive accumulation of glomerular and tubulointerstitial ECM proteins as well as increased GFR, suggesting that deficiency of AT_2_R might accelerate the progress of development of DN. 

There are several possible mechanisms by which deficiency of AT_2_R accelerates the development of DN. First, renal hyperfiltration (a key component causing endothelial dysfunction and mesangial stretch) with increased GFR is a hallmark of early DN [32]. As compared with WT animals, there was pronounced hyperfiltration in AT_2_RKO mice in diabetes; we speculate that this apparent hyperfiltration contributes to the development of hypertension and DN observed in AT_2_RKO mice—with or without diabetes. However, our data contrast with the report by Sourris et al. [33] who claimed that lower GFRs were seen in diabetic AT_2_RKO mice. These disparate results could be explained by technical differences in how the studies were performed: (1) we directly measured the GFR, and the other group of authors used urinary albumin excretion rates (AER) and creatinine clearance to estimate GFR; (2) our animals were diabetic for four weeks, whereas theirs had diabetes for 24 weeks. Thus, the stage of DN was not comparable. Jerums et al. [32] recently reviewed the assessments of AER and GFR in diabetic nephropathy, indicating that changes in AER are dynamic, whereas changes in GFR are usually progressive; additionally increases in AER generally, precede a decline in GFR [32]. 

Second, Rüster et al. [34] indicated that Ang II via AT_2_R in podocytes upregulates RAGE (receptors for AGEs) production, the key pathogenic factor in diabetic renal injury. Recently, Sourris et al. [33] reported that overexpression of RAGE in primary mesangial cell cultures via an adenovector-based construct enhanced superoxide generation with a significant decline of AT_2_R expression; AT_2_RKO mice exhibited significantly higher renal superoxide production in freshly minced kidney cortex as compared with those from WT animals; however, elevated renal superoxide production in the AT_2_RKO remained unaffected by diabetes. Then, they postulated that the pathogenic interactions among the RAGE, AT_2_R deficiency, and ROS generation might impact the development of diabetic renal injury. 

Previously, we demonstrated that intrarenal RAS activation and high glucose via ROS generation may act in concert to increase proximal tubular cell (RPTs) apoptosis and tubulointerstitial fibrosis in diabetes, independent of systemic hypertension [16, 17]. In the current study, we observed that ROS generation in the renal cortex was significantly augmented in RPTs of AT_2_RKO mice as assessed by HO-1 expression, and we validated our observation in freshly isolated RPTs from AT_2_RKO mice. This elevation of ROS was even more pronounced in the kidneys of diabetic AT_2_RKO mice and attenuated in the insulin-treated animals. 

NADPH oxidase plays an important role in the ROS generation in DN. NADPH oxidase consists of several membrane-bound subunits (gp91phox, nox, and p22phox) and cytosolic subunits (p47phox and p67phox). It has been reported that oxidative stress in the diabetic nephropathy is mediated, at least in part, through p47phox activation [35, 36]. By qPCR analysis, we have found that as compared with control WT mice, p47phox mRNA seems to be upregulated in RPTs of AT_2_RKO mice, and this upregulation appears to be more profound in diabetic condition. Taken together, these data suggested that AT_2_R deficiency increased intrarenal ROS generation in RPTs, leading to acceleration of the progress of DN. 

Third, while functional linkage of ACE2-Ang-(1-7)-Mas axis and oxidative stress is evident in the heart [37], the role of ACE2 expression and high glucose/Ang II-induced ROS generation in kidney pathophysiology is still not established [37–39]. To date, evidence indicates that recombinant human ACE2 prevents Ang II-induced hypertension, renal oxidative stress, and tubulointerstitial fibrosis [40] while attenuating diabetic kidney injury in Akita mice in association with reduced blood pressure and decreased NADPH oxidase activity [41]. Since AT_2_R inhibits ACE activity [15], which may, in part, underlie AT_2_R increasingly recognized attenuation of AT_1_R-mediated actions, on the other hand, we speculate that AT_2_R might increase ACE2 activity and then decrease the ACE/ACE2 ratio to prevent the diabetic renal injury. Our data from this study further tested and support this hypothesis in an *in vivo* model.

The key components of the intrarenal RAS, including Agt, AT_1_R, and ACE are significantly upregulated, whereas ACE2 is downregulated, in renal proximal tubules of AT_2_RKO mice compared to WT animals. Additionally, the elevation of AT_1_R, as seen in small intrarenal vessels, also favors the development of DN. Moreover, the increased classic RAS gene expression (i.e., Agt, AT_1_R, and ACE) was further enhanced in diabetic AT_2_RKO mice, and that expression is particularly attenuated in the kidneys of insulin-treated animals. Taken together, these data suggest that intratubular Ang II is increased in proximal tubules of AT_2_RKO with or without diabetes; the increased ACE/ACE2 ratio might accelerate, at least in part, favoring the development of DN.

In summary, our results suggest that deficiency of AT_2_R that enhanced or accelerated the development of diabetic nephropathy is mediated, at least in part, via elevated ROS generation and activation of intrarenal RAS genes with downregulation of ACE2 gene expression in RPTs. 

## Abbreviations

ACE: Angiotensin-converting enzyme
ACE2: Angiotensin-converting enzyme2
AER: Albumin excretion rate
Agt: Angiotensinogen
Ang II: Angiotensin II
AT_1_R and AT_2_R: Ang II receptors type 1 and 2
AT_2_RKO: AT_2_R knockout
BP: Blood pressure
BW: Body weight
DN: Diabetic nephropathy
ECM: Extracellular matrix
FITC: Fluorescein isothiocyanate
GFR: Glomerular filtration rate
HO-1: Heme oxygenase-1
IHC: Immunohistochemistry
KW: Kidney weight
PAS: Periodic Acid Schiff
RAGE: Receptors of AGEs
RAS: Renin-angiotensin system
ROS: Reactive oxygen species
RPTs: Renal proximal tubules
RT-qPCR: Real-time-quantitative polymerase chain reaction
SBP: Systolic blood pressure
STZ: Streptozotocin
TUNEL: Transferase-dUTP-nicked-end labeling
WT: Wild type.
